# Laser-assisted Icon and clinpro for restoring white spot lesions: an in vitro comparative study

**DOI:** 10.1007/s10266-024-01026-9

**Published:** 2024-11-26

**Authors:** Yomna Said Mohamed, Mohamed Shamel, Sara El Banna

**Affiliations:** 1 Department of Pediatric Dentistry, Faculty of Dentistry, Newgiza University, Giza, Egypt; 2 Department of Oral Biology, Faculty of Dentistry, The British University in Egypt, El Sherouk City, Egypt; 3 Department of Oral Biology, October University for Modern Sciences and Arts, 6th October City, Egypt; 4 Dental Science Research Group, Health Research Centre of Excellence, The British University in Egypt, El Sherouk City, Egypt

**Keywords:** White spot lesion, Clinpro XT varnish, Icon resin, Color change, Microhardness, Diode laser

## Abstract

Managing white spot lesions (WSLs) remains a challenging issue that has yet to be fully resolved. WSLs are areas of demineralized enamel that most commonly occur following orthodontic treatments. They can potentially lead to enamel caries and are also esthetically undesirable. The current study investigated and analyzed the effects of Icon resin infiltration (Icon) and Clinpro XT varnish (Clinpro), both alone and in combination with a diode laser, on the restoration of WSLs. Color change, microhardness, and scanning electron microscopy were used to evaluate the WSLs after the different treatment applications. Results showed that the combination of Icon and Clinpro, along with a diode laser, enhanced color stability and restoration of enamel hardness in white spot lesions. Utilizing a diode laser significantly improved the efficacy of both Icon and Clinpro therapies. SEM examination verified that laser-assisted treatments resulted in almost total blockage of enamel rods, indicating enhanced effectiveness. Conclusions: Integrating diode laser treatment with Icon and Clinpro XT Varnish has significantly improved the esthetic outcomes and mechanical properties of treated enamel.

## Introduction

Esthetics is pivotal in dentistry and dental education, with advances in dental technology presenting numerous alternatives that simplify esthetic dental treatments. Patients aspire to achieve a perfect smile after fixed orthodontic treatment. However, due to poor oral hygiene, which can lead to plaque accumulation on smooth tooth surfaces, nearly half of those receiving such treatments develop clinically noticeable white spot lesions (WSLs) [1, 2]. These lesions are opaque, white areas of demineralization located below the outermost enamel layer. WSLs pose significant clinical challenges during orthodontic care due to their rapid development, difficulty in maintaining oral hygiene around brackets, and the esthetic and structural impact they have on teeth. The presence of orthodontic appliances exacerbates plaque accumulation, making WSLs harder to manage and increasing the risk of enamel damage, which is often difficult to reverse [3, 4].

The formation of WSLs is attributed to the loss of minerals caused by organic acids produced by pathogenic bacteria, which dissolve calcium and phosphate ions from the enamel. This process may or may not be reversible through remineralization. Given the tooth structure’s integrity and natural remineralization ability, managing incipient carious lesions like WSLs is crucial. Yet, currently, there is no definitive protocol for managing these lesions. Typically, the initial line of treatment involves remineralization strategies, such as applying calcium phosphate pastes with casein phosphopeptide, bioactive glass (calcium sodium phosphosilicate), and fluoride treatments. Alternative therapies include bleaching, microabrasion, traditional bonding, and various types of veneers [5].

A novel approach introduced recently involves using a low-viscosity resin known as Icon resin infiltration (Icon) for the minimally invasive treatment of WSLs. This method, called resin infiltration technique (RIT), employs a 15% HCl acid etch to create enamel porosity, allowing for the infiltration of a highly viscous and deeply penetrating resin [6]. This treatment not only halts the progression of WSLs but also strengthens the area against further cariogenic attacks and conceals the typical opaque white appearance by matching the refractive index of enamel [7].

On the other hand, Clinpro XT varnish (Clinpro) is a resin-modified glass ionomer cement developed to restore WSLs. Its primary mode of action is mediated by the liberation of fluoride, which facilitates the remineralization process in demineralized enamel and diminishes its solubility in acidic circumstances [8, 9]. Fluoride ions facilitate the generation of fluorapatite, a substance with lower solubility than hydroxyapatite, augmenting the enamel’s ability to withstand acid erosion [10, 11].

Despite these advancements, only a few in vitro studies over the past two decades have investigated the effects of combining remineralizing agents with diode laser irradiation on enamel to treat WSLs. These studies suggest that such combination therapies result in greater fluoride uptake and increased enamel hardness compared to fluoride-only treatments [12–15]. Moreover, one unique study demonstrated that this combination therapy significantly improved the hardness and esthetic appearance of WSLs on bovine teeth [16]. However, whether diode laser irradiation combined with resin infiltration can enhance the esthetic appearance and hardness of WSLs remains unproven.

A notable gap exists in the comparative analysis of emerging treatments, particularly the combined use of novel resin infiltration techniques and laser-assisted therapies. Previous studies have primarily focused on individual treatment outcomes, leaving a critical knowledge gap regarding the synergistic effects of combined treatment modalities on both the esthetic and mechanical properties of enamel post-WSLs.

Therefore, this in vitro laboratory study is specifically designed to evaluate the effectiveness of Icon and Clinpro, when combined with diode laser irradiation, in enhancing the esthetic qualities and microhardness of artificial WSLs. The null hypothesis is that there is no significant difference in esthetic improvement (color stabilization) or mechanical enhancement (microhardness) of artificial white spot lesions (WSLs) when treated with the combined use of diode laser irradiation with Icon and Clinpro compared to using Icon or Clinpro alone.

## Materials and methods

### Study design

After obtaining informed consent, 32 premolar teeth were collected from patients undergoing orthodontic treatments. The teeth were sound, without any signs of restoration, decalcification, or apparent caries. The sample size was determined using power analysis where *n* = 2(1.96 + 0.84)^2^(2)^2^/(1)^2^ ≈32. The study was conducted following ethical approval (REC-77723). An ultrasonic scaler and slurry pumice were used to clean the teeth thoroughly and remove any remaining soft tissues, calculus, and plaque before storing them in normal saline for the study. All samples were randomly allocated to four groups where each tooth was sectioned in a mesiodistal direction into equal halves, and each half was divided into two halves. Each quarter was embedded in chemically activated acrylic resin to expose the enamel. Four groups were prepared as per treatment modality:

- Group A: Clinpro
- Group B: Icon
- Group C: Clinpro + diode laser

- Group D: Icon + diode laser

### Specimen preparation

All samples were demineralized by being immersed and stored in a demineralizing solution (2.2 mM calcium chloride, 2.2 mM monopotassium phosphate, 0.5 mM acetic acid with pH adjusted to 4.4 and 1 M potassium hydroxide) for 96 h to produce a WSL. All the teeth were thoroughly washed with distilled water and then air-dried [17].

The teeth of groups A and C were etched using 37% phosphoric acid for 30 s. Subsequently, they were rinsed with water and left to air-dry for 3 s. Next, a thin coat of Clinpro (3 M ESPE, Pymble, New South Wales, Australia) was administered and exposed to light curing for 20 s [18].

For the Icon resin application (groups B and D), the teeth were treated with a 15% hydrochloric acid gel (Icon-Etch, DMG) for 2 min. They were washed with water and dried using moisture-free air for 30 s. A solution of 99% ethanol (Icon-Dry, DMG) was used, which was applied for 30 s and then allowed to dry in the air. Icon (DMG America, Englewood, NJ, USA) was applied and left to sit for 3 min, after which it was subjected to light curing for 40 s. A second layer of Icon was administered, allowed to sit for 1 min, and then exposed to light cure for 40 s [18].

### Diode laser protocol

A commercially available diode laser (Gallium–Aluminum–Arsenide [Ga AIAs] diode laser, Simpler, Doctor Smile, Italy) of 980 nm wavelength and power output of 2 W was used in a continuous mode. Following the application of the materials, the laser was immediately applied to the prepared tooth surfaces for a duration of 15 s per specimen. It was attached to a 220-micron optic conductor fiber as a transmission element. The optic fiber was moving uniformly and longitudinally over the treated tooth surface by the same operator for all the specimens, ensuring consistent application across the study [19].

### Color measurement

The color of each sample was assessed at three time points: baseline before WSL, after WSL, and after the application of each of our study materials. The apparatus used in measurements is the Cary 5000 Spectrophotometer provided by Agilent Technologies (USA). Agilent Cary 5000 UV–Vis–NIR spectrophotometers are manufactured using a quality management system certified to ISO 9001.

The measurements were conducted in the same examination room with standardized lighting conditions. To guarantee precision, the spectrophotometer was calibrated before each test using a white calibration plate supplied by the manufacturer. This calibration accounted for any discrepancies in the illumination output from the internal light source. The spectrophotometric data of each tooth were obtained through two separate measurements. This involved placing the measuring head on the tooth’s surface, removing it, and then repositioning it. Consequently, the device produced a definitive numerical value for each tooth, resulting in accurate readings. The color measurements were quantified using the coordinate values L*, a*, b* specified by the Commission Internationale de l’Eclariage, 1978 (CIE) [20].

### Microhardness measurement

The microhardness measurements were taken at three randomized indentations on the treated enamel surface of each sample. The indentations were made approximately 0.3 mm away from each other using a Vickers Microhardness Tester (Model HVS-50, Laizhou Huayin Testing Instrument Co., Ltd. China) with a Vickers diamond indenter and a 20× objective lens. Each measurement used a 200-g load for 10 s, oriented perpendicularly to the tooth surface [21].

### Scanning electron microscope (SEM)

All samples were examined under a scanning electron microscope (SEM; Model FEI Quanta 3D 200i). Samples were fixed on aluminum stubs with standard diameter using a carbon double sticky tape attached to the EDX Unit (Energy-Dispersive X-ray Analyses/Thermo Fisher pathfinder) that was employed to investigate the morphological structures and elemental composition of the samples under operating conditions of accelerating voltage 30 K.V, resolution for Gun.1 nm, and magnification × 2000 and × 3000 [22]. Images were gathered at the following time points: baseline, after WSL, and after applying the study materials.

### Statistical analysis

The color change and microhardness results are presented as means ± standard deviations. The Shapiro–Wilk test was used to assess the normality of the data. A one-way ANOVA with Tukey’s post hoc test were used to determine the statistical significance between the experimental groups. A value of p < 0.05 was considered significant. GraphPad Prism 9.0 (GraphPad Software, San Diego, CA, USA) was used for the statistical analysis.

## Results

### Color change

**Fig. 1:**
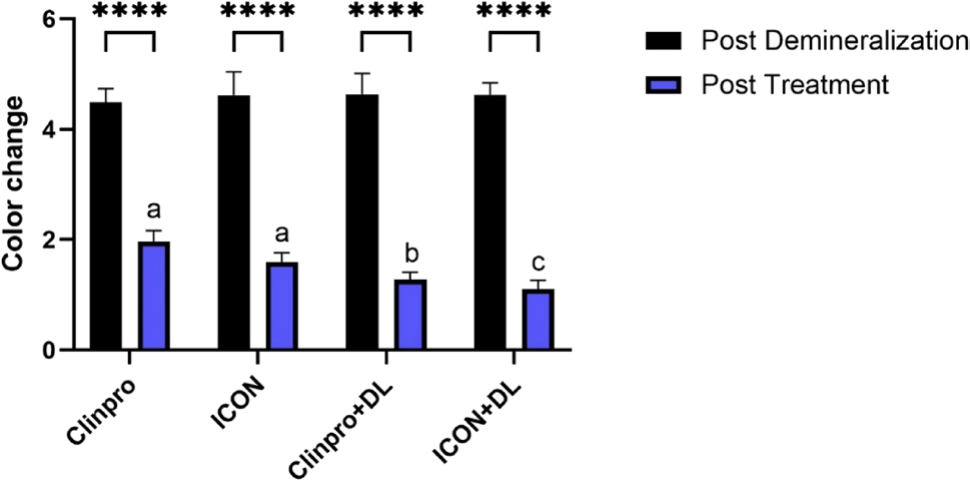
Mean and standard deviation of the color change of experimental groups compared to baseline post-demineralization and post-treatment. Different letters indicate significance

L, a, and b values of the CIE color measurements of the samples were measured at three time intervals: baseline, post-demineralization, and post-treatment (Table 1). Color change compared to the control (pre-demineralization) was calculated, and it showed that post-demineralization, all groups had a significant (*p* < 0.05) color change, indicating the formation of white lesions. Further color change post-treatment was significant (*p* < 0.05) in all groups when compared to their post-demineralization state, highlighting the effectiveness of the treatments in altering color. The Icon + DL and Clinpro + DL treatments led to the slightest color change, suggesting their superiority in maintaining closer to baseline color. No significant difference (p0.7) was found between Icon + DL and Clinpro + DL, nor between Clinpro and Icon (*p* = 0.2), indicating similar efficacy in color stabilization between these treatments (Fig. 1).

**Table 1:** The experimental groups’ *L*, *a*, and *b* color values at baseline, post-demineralization, and post-treatment. Color change differences between baseline, post-demineralization, and post-treatment values are shown

Group	CIE lab values									Color differences between baseline and post-demineralization				Color differences between baseline and post-treatment			
	Baseline			post-demineralization			post-treatment										
	L*	a*	b*	L*	a*	b*	L*	a*	b*	Δ*L*	Δ*a*	Δ*b*	Δ*E*	Δ*L*	Δ*a*	Δ*b*	Δ*E*
**Clinpro**	64.2	1.2	14.1	67.7	0.4	11.5	62.6	1.3	15.3	3.6	−0.8	−2.6	4.5	−1.6	0.2	1.2	2.0
**Icon**	66.1	1.1	13.5	69.9	0.4	10.9	64.8	1.3	14.4	3.7	−0.7	−2.6	4.6	−1.3	0.2	0.9	1.6
**Clinpro + DL**	66.5	1.2	13.3	70.2	0.4	10.7	65.4	1.3	13.9	3.8	−0.8	−2.6	4.6	−1.1	0.1	0.6	1.3
**Icon + DL**	67.1	1.5	12.9	70.6	0.8	10.1	66.1	1.6	13.4	3.6	−0.7	−2.9	4.6	−1.0	0.1	0.5	1.1

### Microhardness

**Fig. 2:**
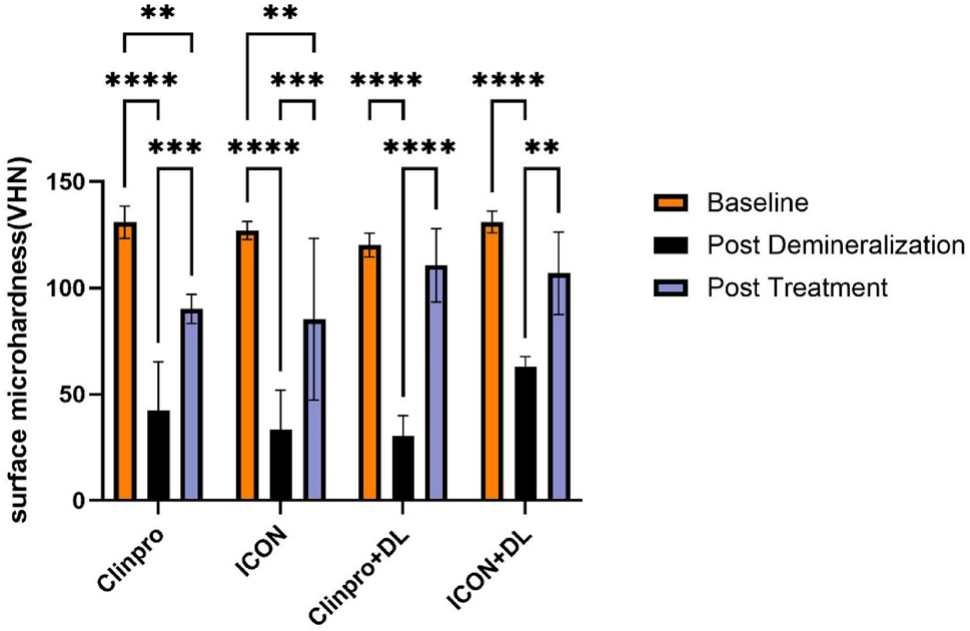
Mean and standard deviation of surface microhardness (VHN) of all experimental groups at baseline, post-demineralization, and post-treatment. *p < 0.05, **p < 0.01, ***p < 0.001, **** *p* < 0.0001 indicates significance

The surface microhardness measurements showed a significant decrease (*p* < 0.05) post-demineralization across all groups, evidencing the damaging effect of demineralization on enamel hardness. Results showed that the surface microhardness of all groups after demineralization significantly decreased (*p* < 0.05) compared to the baseline values. Post-treatment analysis revealed a non-significant difference in microhardness values comparing Clinpro + DL (*p* = 0.7) and ICON + DL (*p* = 0.1) treatments with the baseline values. These treatments resulted in the highest microhardness values, closely matching or slightly deviating from baseline levels, indicating effective restoration or preservation of material hardness. Conversely, significant lower microhardness values were found when treatments with ICON (*p* = 0.003) and Clinpro (*p* = 0.003) were used alone, suggesting a lesser degree of recovery or protection offered by these treatments (Fig. 2).

### Scanning electron microscope (SEM)

**Fig. 3:**
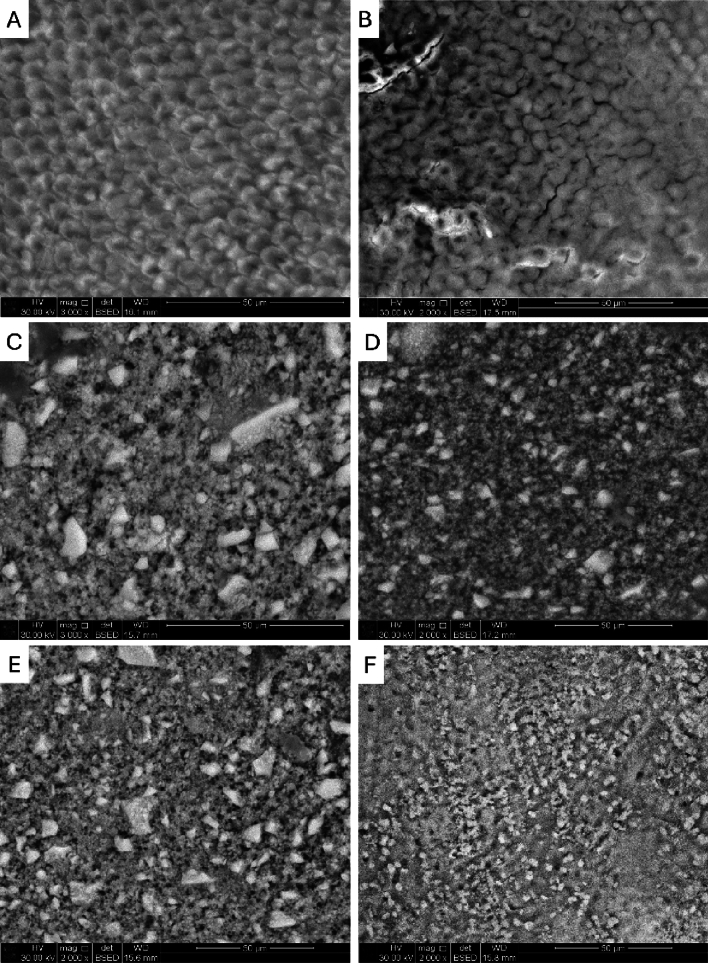
SEM images of the control group (**A**) revealed intact enamel with a smooth surface and enamel rods that were arranged in a typical manner. The inter-rod substance created a distinctive keyhole pattern (magnification × 3000). The etched enamel surface (**B**) displays irregularities and various porosities, destroying the interprismatic substance and creating multiple cracks (magnification × 2000). SEM images of the enamel surface after application of Clinpro (**C**) and ICON (**D**) showed partial obstruction of the enamel rods due to resin infiltration, resulting in an irregular and rough topography. Observe the existence of a mineralized layer that entirely coated the surface of the enamel (magnification × 2000). SEM images of the enamel surface after Clinpro and Laser (**E**) and ICON and Laser (**F**) showed nearly complete obliteration of the rods (magnification × 2000)

SEM images of the untreated enamel surface showed intact enamel with a smooth surface and enamel rods that were arranged in a typical manner. The inter-rod substance created a distinctive keyhole pattern (Fig. 3A). After the acid etching, enamel lost its normal features with the destruction of rods and the appearance of cracks and pores (Fig. 3B). After Clinpro and ICON, the enamel surface showed partial occlusion of the enamel rods by mineralized deposits of varying sizes and shapes (Fig. 3C, D). After applying the laser with Clinpro and ICON, the enamel surface showed nearly complete obstruction of the enamel rods (Fig. 3E, F).

## Discussion

This study uniquely analyzed the effects of resin infiltration (Icon) and Clinpro, both individually and in combination with diode laser treatment, on white spot lesions (WSLs) in vitro. The resin infiltration technique employed by Icon is distinct in its approach, which targets the internal structure of the lesion, thus offering a durable restoration solution without the need for invasive procedures. Meanwhile, Clinpro, combined with sodium fluoride and calcium phosphate embedded in a resin carrier, provides dual-action remineralization and surface protection against acidic challenges. In recent years, diode lasers have become widespread in various fields of dentistry [23, 24]. Studies showed that low-power red and near-infrared lasers can effectively prevent caries [25, 26]. However, laser light must be absorbed and transformed into heat without causing harm to underlying tissues. In this way, laser light can successfully alter the solubility and composition of dental hard tissues [27]. Notably, this study has demonstrated that combining these treatments with diode laser irradiation enhances the enamel’s esthetic and mechanical properties, a significant advancement over traditional methods that do not combine these modalities [28].

In the current study, the observed modification in color after demineralization in all experimental groups provided evidence for the effectiveness of the treatment in forming white spot lesions. The observed post-treatment outcomes, which demonstrate a notable improvement in color compared to the initial state, suggest that both therapies effectively reduce the visual consequences of white spot lesions. All experimental groups showed statistically significant differences compared to their baseline, proving that each treatment efficiently improves the WLS. This is similar to previous studies` findings, which showed that resin infiltration produced favorable esthetic results [18, 29]. However, both Icon with DL and Clinpro with DL had comparable results and effectiveness. They were better than those used without DL, which implies that incorporating laser treatment could potentially augment the efficacy of both Icon resin and Clinpro. It is worth mentioning that there was no substantial disparity in color stabilization across the therapies, underscoring their similar efficacy in this aspect. This is in accordance with the study by Alqahtani et al. [30] that concluded fluoride combination therapy significantly improved the esthetic appearance of WSLs compared to the fluoride-only group. Although it wasn’t statistically significant, Clinpro showed more color improvement rather than Icon treatment alone, which was not similar to previous studies that concluded that icon infiltration had significantly better results than Clinpro treatment [18, 31].

The observed color differences, while statistically significant, must also be considered in the context of clinical relevance. In clinical practice, the primary goal of treating WSLs is to achieve an esthetic outcome that is satisfactory to the patient and indistinguishable from the surrounding enamel. The color improvement observed in this study although quantitatively measured, reflects a noticeable visual enhancement that would be perceptible to both clinicians and patients.

The observed reduction in surface microhardness following demineralization suggests that demineralization negatively impacts enamel hardness, a critical determinant of teeth’s vulnerability to subsequent decay and mechanical abrasion. The analysis conducted after treatment indicated that therapies utilizing laser assistance, specifically Clinpro + DL and Icon + DL, demonstrated the highest efficacy in restoring or preserving enamel microhardness. This observation implies that the utilization of laser treatment might improve the penetration depth and activation of resin infiltration and varnish, resulting in a more resilient re-hardening of the enamel. The lower microhardness values observed with Icon and Clinpro treatments alone could indicate that these treatments, while beneficial, may not be as effective in recovering enamel hardness to its baseline level without the adjunctive use of laser treatment**.** This concurs with the study conducted by Moharam et al. [19], who concluded that diode laser combined with fluoride therapy showed the highest significant surface microhardness. However, this finding was not similar to Alqahtani et al. [30], who concluded that fluoride combined diode laser had less enamel hardness versus treatment with fluoride only.

SEM of the untreated enamel surface showed intact enamel with a smooth surface and enamel rods that were arranged in a typical manner. After Clinpro and Icon, the enamel surface showed partial occlusion of the enamel rods by mineralized deposits of varying sizes and shapes. After applying the laser with Clinpro and Icon, the enamel surface showed nearly complete obstruction of the enamel rods, proving that incorporating laser treatment improves the effectiveness of Icon resin and Clinpro.

In summary, the current study provides valuable information on managing WSLs. The analysis suggests that combining laser treatment with either Icon or Clinpro significantly enhances treatment outcomes regarding color stability and enamel hardness. The study’s comparative assessment of these treatments offers valuable insights into their individual effectiveness and potential synergistic effects, particularly when combined with laser treatment to improve treatment results.

One of the key limitations of this study is its in vitro nature. While in vitro studies provide a controlled environment to test hypotheses and observe effects, they do not fully replicate the complex conditions present in the oral cavity. Factors, such as saliva flow, the presence of biofilm, variations in oral hygiene practices, and patient-specific behaviors, which can significantly influence the outcomes of treatments in a clinical setting, are not accounted for in an in vitro model. Therefore, further in vivo studies are necessary to validate these findings and assess their applicability.

## Conclusion

Integrating diode laser treatment with Icon and Clinpro has significantly enhanced the esthetic outcomes and mechanical properties of treated enamel.

## Clinical significance

The clinical implications indicate that combining diode laser with Clinpro or Icon can be a noninvasive method for enhanced improvement of white spot lesions. This method can lessen the need for invasive operations while simultaneously increasing patient satisfaction, enamel strength, and cosmetic results. Additional research could improve these protocols and confirm their long-term efficacy.

## Data Availability

The datasets analyzed during the current study are available from the corresponding author upon request.
